# Catheter ablation superiority over the pharmacological treatments in atrial fibrillation: a dedicated review

**DOI:** 10.1080/07853890.2021.1905873

**Published:** 2021-03-30

**Authors:** Abdullah Alrumayh, Muath Alobaida

**Affiliations:** Department of Basic Sciences, King Saud University, Riyadh, Saudi Arabia

**Keywords:** Catheter ablation, atrial fibrillation, cardiovascular disease, interventional cardiology

## Abstract

Atrial fibrillation globally affects roughly 33.5 million people, making it the most common heart rhythm disorder. It is a crucial arrhythmia, as it is linked with a variety of negative outcomes such as strokes, heart failure and cardiovascular mortality. Atrial fibrillation can reduce quality of life because of the potential symptoms, for instance exercise intolerance, fatigue, and palpitation. There are different types of treatments aiming to prevent atrial fibrillation and improve quality of life. Currently, the primary treatment for atrial fibrillation is pharmacology therapy, however, these still show limited effectiveness, which has led to research on other alternative strategies. Catheter ablation is considered the second line treatment for atrial fibrillation when the standard treatment has failed. Moreover, catheter ablation continues to show significant results when compared to standard therapy. Hence, this review will argue that catheter ablation can show superiority over current pharmacological treatments in different aspects. It will discuss the most influential aspects of the treatment of atrial fibrillation, which are recurrence and burden of atrial fibrillation, quality of life, atrial fibrillation in the setting of heart failure and mortality and whether catheter ablation can be the first line treatment for patients with atrial fibrillation.

## Introduction

Atrial fibrillation (AF) is the most common cardiac arrhythmia, with substantial morbidity and mortality [1]. As an estimation, more than 10% of the population will have developed AF by age 75 [2]. AF mortality usually results from, but is not limited to, cardiovascular death, heart failure (HF) or cardioembolic/ischemic stroke [3]. Currently, the main aim of AF treatment is to improve quality of life (QoL) and prevent morbidity and mortality related to AF. Unfortunately, AF treatment can be challenging and complex because the available treatment options have possible side effects, and there is no guarantee of curing AF with all the available treatments. The treatment of AF, according to the present guidelines, encourage the use of pharmacology interventions as the first line treatment for patient without left ventricular dysfunction or selected patient with heart failure reduced ejection fraction [4]. Symptomatic patients with AF may have an intolerance for antiarrhythmic medication, or the medication may be ineffective to restore normal sinus rhythm, which indicates that the patient should undergo, as a second line treatment, catheter ablation. The discovery of a key development in understanding AF in observed electrical triggers of AF in the pulmonary veins led to pulmonary vein isolation (PVI) using catheter-based intervention to treat AF [5]. In addition, since catheter ablation demonstrated a high successful rate of AF reduction [6–9], it has progressed into an interventional option, which treats a wide variety of symptomatic AF patients. Presently, radiofrequency catheters and cryoballoon catheters are the most common devices to perform PVI to treat AF with no significant difference in terms of safety or efficacy [10–13]. However, catheter ablation, indeed, poses serious complications (e.g. stroke, cardiac tamponade, and pulmonary vein stenosis 14] that are often more urgent and dramatic than those with pharmacology therapy [15–17]. Despite that, catheter ablation, when performed in experienced centres and by a skilled electrophysiologist, can be successful and safe for a majority of patients [3]. This highlights the importance of patient selection and clinician experience. This review will explore the effectiveness of catheter ablation when compared to standard therapy in regard to the recurrence and burden of AF, QoL, mortality, and AF in the setting of HF patients by looking at the most recent and most influential trials.

### The burden and recurrence of atrial fibrillation

Restoring and maintaining a normal sinus rhythm is a crucial element in the management of AF. In some cases, it is well-known that one of the approaches in the management of atrial fibrillation is restoration of sinus rhythm, although ventricle rate control might be effective in some patient but requires anticoagulation medications that poses risk of bleeding [18]. Currently, according to ESC guideline, catheter ablation of AF may restore and sustain sinus rhythm in symptomatic patients with all AF types (i.e. paroxysmal, persistent, and possibly long-standing persistent AF) when the standard treatment (i.e. antiarrhythmic medication therapy) has failed [5,19]. AF catheter ablation, as reported by several randomised clinical trials [14,20–24], provide a significant restoration and management of sinus rhythm compared to antiarrhythmic drugs. However, some of these trials, such as the ThermoCool AF trial and STOP AF [20,24], were recruiting participants who failed the first line treatment of AF. Consequently, it is difficult to define superiority in these trials, rather than supporting the guidelines. Further, short and long-term trials (MANTRA-PAF [6,25] and RAAFT-2 [7]) attempt to investigate the superiority in naïve patients with paroxysmal AF (i.e. not refractory to antiarrhythmic drug treatment). Although these trials have some limitations such as a focus on, mainly, a low-risk patients, limited age group (<70), small sample size, and performed in a highly experienced centres, they show a significant improvement of catheter ablation over the standard treatment in the context of recurrence and burden of AF. Moreover, the recent large and long-term trial CABANA, by Packer et al. [3], contributed valuable discoveries to the practice of catheter ablation, showed a significant low rate of AF recurrence (adjusted HR, 0.52 [95% CI, 0.45–0.60]; *p* < .001) over 48 months for catheter ablation against state-of-the-art pharmacology therapy. The CABANA trial managed to overcome some of the limitations that the previous related trials experienced by using a large sample size (*n* = 2204) and including participants with different types of AF (persistent and long-standing persistent AF). Generally, these trials demonstrate that catheter ablation has better outcomes in terms of AF recurrence and for some AF burden (Table 1). However, catheter ablation is still not curative. Additionally, and to be relevant, 17.1% of patients in the CABANA trial had to undergo ablation for a second time. The pathophysiological reason behind the first onset of AF may contribute to its recurrence even with an initially successful ablation. Further investigations with regard of risk factors management necessary in order to examine the recurrence frequencies. In addition, to ensure accuracy in the measurement the AF burden, future studies need to use continuous rhythm monitoring using implantable cardiac rhythm monitors rather than 7-day Holter monitoring.

**Table 1.** CA vs pharmacology treatment in AF (the burden and recurrence and QoL of AF).

Setting	Trials	Sample size	Duration (mo)	Primary outcomes (CA arm)	Superiority (CA arm)
The burden and recurrence of AF	ThermoCool AF [20] (2010)	167	9	HR: 0.03 (95% CI, 0.19–0.47; *p* <.001)	Superior
	MANTRA-PAF [25] (2017)	294	24	90th percentile, 9% vs.18%; *p* = .007	Superior
	RAAFT-2 [7] (2014)	127	24	HR: 0.56 (95% CI, 0.35–0.90; *p* <.02)	Superior
	Early-AF [23] (2021)	303	12	HR: 0.48 (95% CI, 0.35–0.66; *p* <.001)	Superior
	STOP AF [24] (2013)	245	12	*p* < .001	Superior
	CABANA [3] (2019)	1240	48	HR: 0.52 (95% CI, 0.45–0.60; *p* <.001)	Superior
Quality of life	MANTRA-PAF [25] (2017)	294	24	*p* < .15	Non-inferior
	RAAFT-2 [7] (2014)	127	12	*p* < .25	Non-inferior
	Cryo-FIRST [30] (2020)	220	12	(95% CI: 5.5–14.2; *p* < .0001)	Superior
	CABANA [29] (2019)	2204	12	(95% CI: −2.0 to −1.1; *p* < .001)	Superior
	CAPTAF [31] (2019)	155	12	(95% CI: 3.1–14.7; *p* < .003)	Superior

CA: Catheter ablation; AF: atrial fibrillation; QoL: quality of life; mo: months; HR: hazard ratio; CI: confident interval.

### Quality of life

The treatment with catheter ablation does not necessarily guarantee an absolute cure for AF. Although catheter ablation for AF has become less certain, attention has been directed to the benefits of catheter ablation on QoL associated with AF [26]. Symptoms such as fatigue, palpitations and limited exercise tolerance caused by AF may significantly reduce the patient’s QoL [27]. Interpretation of QoL results can be difficult because there is a chance of bias due to the lack of blinding; and as commonly seen in clinical trials, cross-over between groups, and lastly the measures used are usually unfamiliar to both practitioners and patients [28]. Some trials [6,7,25] have attempted to investigate the effects of catheter ablation on naïve patients with paroxysmal AF, as compared to pharmacology therapy. Yet, even though there was significant improvement of QoL groups, both of the trials failed to show a significant improvement for the catheter ablation arm over the standard treatment. These studies, however, have limitations such as small participant size and primarily include patients with paroxysmal AF only. Long-term follow-up trials include patients with different types of AF is required to evaluate the QoL. The co-primary end-point for CABANA, by Mark et al. [29], was mainly focussed on the QoL, which can draw a better conclusion than the previous smaller trials. CABANA used two different scores to measure the QoL Atrial Fibrillation Effect on Quality of Life (AFEQT) and the Mayo AF-Specific Symptom Inventory (MAFST). There was a significant improvement of QoL of catheter ablation over standard treatment (adjusted difference, 5.3 points [95% CI, 3.7–6.9]; *p* < .001; for AFEQT) and (adjusted difference, −1.7 points [95% CI, −2.3 to −1.2]; *p* < .001; for MAFSI frequency). Similarly, the Cryo-FIRST and CAPTAF trial [30,32] shows similar significant results as CABANA [29] with regard of QoL for patients who are refractory to antiarrhythmic medications, making these findings more robust. These findings show a superiority of catheter ablation over pharmacological therapy and can assist with decision-making for managing AF, see Table 1.

### Catheter ablation for heart failure patients

Heart failure (HF) and AF are mutual coexisting conditions [32,33], with AF increasing the risk of hospitalisation for HF and death [4,34,35]. The underlying pathophysiology of AF and HF may be intervolved [36]. Long-term uncontrolled AF often leads to tachycardia-induced cardiomyopathy which result in noticeable reduction in ejection fraction (EF) with increase HF deterioration. Moreover, long-term HF may possibly increase the risk of developing AF after the increase of left atrial size and left atrial fibrosis [37]. There is still a lack of knowledge as to why there is a high mortality rate of HF patients with persistent AF. Although the treatment of AF can considerably change long term results with HF, the most effective management plan is still debateable. Many studies [37–40] elaborate that catheter ablation is linked to favourable results in patients with HF. Nevertheless, there is still an ongoing debate between rate versus rhythm control in the management of AF with pharmacology therapy [36]. Currently, for HF patients with AF, amiodarone and dofetilide (outside of Europe) are the only recommended antiarrhythmics medications [4]. These medications are usually discontinued early because of the drug-drug interaction and the related side effects. In addition, amiodarone may lead to multi-organ toxicity while dofetilide has a risk of torsades de pointes and contraindicated in patients with renal failure which usually occur alongside HF [32]. Prior observational studies [41,42] illustrated better survival outcomes for HF patients whose sinus rhythm is maintained, which, as discussed earlier, provisionally puts catheter ablation superior to conventional therapy. In the AATAC long-term trial (Ablation versus Amiodarone for Treatment of Atrial Fibrillation in Patients with Congestive Heart Failure and an Implanted ICD) [40], showed significant improvements in the 6-minutes walk test (6MWT), left ventricular function, and Minnesota Living with Heart Failure Questionnaire (MLHFQ) scores in patients who were treated with catheter ablation instead of amoidarone when they did not have recurrence of AF. However, this trial may have caused confusion as to whether ablation is superior to conventional therapy or not, since a majority of the patients in both arms received β-blockers. A recent long-term trial, CASTLE-AF [43], also investigated whether catheter ablation presented better results when compared to pharmacological therapy, demonstrated a substantial reduction of hospitalisation for worsening heart failure in ablation arm compared to pharmacological treatment (37 [20.7%] vs. 66 [35.9%]; hazard ratio, 0.56; 95% CI, 0.37 to 0.83; *p* = .004). However, the trial received criticism because of its extended enrolment time and a comparatively large number of lost follow-up participants between randomisation and end-point of the study. Yet, the results still consider valuable to favour the use of ablation over pharmacological agents. These trials (Table 2), and another trial [44], were not blinded and they measured the EF by echocardiography, which is unreliable for patients with AF as using cardiac magnetic resonance (CMR). Additionally, the target group were patients with minimal to mild HF, whose left ventricle function was only modestly impaired. Therefore, current evidence from long-term trials cannot imply the certainty of ablation superiority over conventional therapy. Nevertheless, these findings, along with short-term trials [38,39,44,45], show that there is superiority and may cautiously extend the implication of catheter ablation to patients with compensated HF with New York Heart Association Functional Classification (NYHA) class III with reduced EF. Correspondingly, the new American college of cardiology and the American heart association (ACC/AHA) focussed update of 2019 support these findings [46].

**Table 2.** CA vs pharmacology treatment in the treatment of AF patient with HF.

Setting	Trials	Sample size	Duration (mo)	Outcomes (CA arm)	Superiority (CA arm)
Heart failure patients	ARC-HF [39] (2013)	52	12	Peak O_2_ consumption: *p* = .018 Minnesota score: *p* = .019 BNP: *p* = .045 SMWT: *p* = .095 EF: *p* = .055	Superior
	AATAC [40] (2016)	203	24	Recurrence free: *p* < .001 Unplanned hospitalisation rate: *p* < .001 Mortality: *p* = .037	Superior
	CASTLE-AF [43] (2018)	133	37.8	Primary composite end point:^a^ HR: 0.62; (95% CI, 0.43–0.87; *p* = .007) Death from any cause: HR: 0.53; (95% CI, 0.32–0.86; *p* = .01) Hospitalized for worsening HF: HR: 0.56; (95% CI, 0.37–0.83; *p* = .004) Death from Cardiovascular causes: HR: 0.49; (95% CI, 0.29–0.84; *p* = .009)	Superior
	CAMTAF [44] (2014)	50	6	LVEF improvement: *p* = .015 Peak O_2_ consumption: *p* = .014 Minnesota score: *p* = .001	Superior
	CAMERA-MRI [45] (2017)	301	6	LVEF improvement: *p* = .0002	Superior

CA: Catheter ablation; AF: atrial fibrillation; HF: heart failure; mo: months; BNP: B-type natriuretic peptide; SMWT: six-minute walk test; EF: Ejection fraction; HR: Hazard ratio; CI: Confident interval; LVEF: Left ventricular ejection fraction.

^a^ The primary end point was a composite of death from any cause or hospitalisation for worsening heart failure.

### Mortality

According to epidemiological studies, AF has been correlated with unfavourable outcomes, including mortality [47,48] and other major cardiac morbidities, such as disabling stroke, congestive HF, and late cognitive impairment [3]. It is unclear to what extent these risks can be reduced by maintaining sinus rhythm. Similar to what was mentioned earlier with regard to managing HF patients with AF, there is ongoing investigation and uncertainty regarding rhythm control versus rate control in the context of mortality reduction [49–54]. Thus, the comparison between catheter ablation and conventional therapy seems reasonable, since it has been proven, as mentioned above, that catheter ablation shows a substantial improvement in restoring sinus rhythm, free of recurrence of AF, and to a certain extent favourable for HF patients. A recent trial, CASTLE-AF [43], along with randomised clinical [40,44] and observational [48] studies and a large registry [55] examined the catheter outcomes with regard to mortality, and they aligned together to show improvement in survival and hospitalisation outcomes. On the other hand, a recently released trial “CABANA” [3] with 2,204 symptomatic patients with different types of AF and average of four years of follow-up, aimed to show superiority for catheter ablation against conventional therapy with regard to primary composite endpoint (mortality, disabling stroke, serious bleeding, or cardiac arrest). The study shows similar results between the two groups in the primary end-point. Moreover, even though there were significant improvements (*p* = .001) in two of the pre-specified secondary outcomes (total mortality or cardiovascular hospitalisation and AF recurrence). For the third pre-specified secondary end-point (all-cause mortality), results failed to show significant variation between the groups. Nevertheless, there was a high rate of crossovers (27.5%) in patients who moved from the drug therapy group to the ablation group which ought to be considered when interpreting the outcomes. Undeniably, catheter ablation did not show better results when it was compared with standard treatment in large scale long-term studies. The previously mentioned trials, CASTLE-AF [43] and AATAC [40], regardless of their small sample size, showed significant improvements in survival of HF patients. Hence, catheter ablation may be superior in terms of survival for a specific group of patients. There is a need to conduct further long-term studies with large samples to examine whether catheter ablation is superior for certain patients (e.g. HF patients) or whether ablation is superior for specific types of AF.

### Catheter ablation as the first-line treatment

In considering the large number of trials that were conducted comparing catheter ablation with standard treatment, catheter ablation has been shown to be superior with different types of outcomes (i.e. recurrence and burden of AF, QoL for HF patients) and non-inferiority (i.e. mortality). Some of these outcomes may indicate that it is more effective to use catheter ablation as the first line of treatment for AF patients. The current evidence supports the finding of favourable results for catheter ablation. However, there are still many limitations to overcome before relying on catheter ablation as a first line intervention. Firstly, the guidelines follow the concept of “first do no harm,” and, undoubtedly, catheter ablation comes with the risk of serious complications (e.g. procedural stroke and pulmonary vein stenosis) [14], which places a significant limitation to the current trials, since they gathered data from an experienced centre with skilled professionals who perform the ablation, while it is notable that in real world practice more junior and less experienced practitioners may perform the procedure [56]. Secondly, there are plethora of trials conducted have limited population eligible for further studies with regard to age, gender, and body mass index (BMI) which may intervene with the presented results. For instance, after catheter ablation, a BMI of ≥30 kg/m^2^ patient have a higher rate of recurrence of AF [57], and women have higher chances of having worse QoL than men [58]. Thirdly, the trials often recruit participants who are relatively healthier than patients with coexisting morbidities, particularly among HF patients. Thus, it needs to be considered that catheter ablation may not be suitable for any patients who meet the criteria. Lastly, some trials show significant results to the ablation arm in the secondary end-points which the sample size may not be powered enough (or centrally adjusted) to describe fully the benefits of catheter ablation in these settings. Therefore, it is important to conduct further studies to examine specific aspects of AF (e.g. recurrence and QoL) as a primary end-point. Hence, catheter ablation still need to be viewed with a cautious approach when making a decision in treating AF. Additionally, ideally, a joint decision between the patient and the cardiologist is vital in determining the type of treatment and decide whether catheter ablation is suitable as a first line treatment.

## Conclusion

Atrial fibrillation is the most common arrhythmia and it can lead to several other comorbidities. The current treatment can not provide a cure to AF rather than help reduce the recurrence of AF and subsequently lead to improve QoL and AF-related morbidities and mortality. The evolution of catheter ablation in time shows a superiority over conventional therapy in regard of recurrence and burden of AF, QoL, and with cautious as a treatment for AF in HF patients. On the other hand, mortality, according to the long-term trial, still shows non-inferiority. Additionally, in regard of treatment plan, catheter ablation still facing some trials limitations needed to be consider before change the recommended guidelines to be the first line treatment. More robust long-term studies with attempts to overcome limitations are needed to support the current outcomes and possibly show certain superiority in regard of mortality.
